# Successful management of severe intrahepatic cholestasis of pregnancy: report of a first Japanese case

**DOI:** 10.1186/1471-230X-14-160

**Published:** 2014-09-13

**Authors:** Kenya Kamimura, Hiroyuki Abe, Naomi Kamimura, Masayuki Yamaguchi, Maiko Mamizu, Kanna Ogi, Yoshifumi Takahashi, Ken-ichi Mizuno, Hiroteru Kamimura, Yuji Kobayashi, Manabu Takeuchi, Kunihiko Yoshida, Kyoko Yamada, Takayuki Enomoto, Koichi Takakuwa, Minoru Nomoto, Miki Obata, Yoshinori Katsuragi, Yukio Mishima, Ryo Kominami, Tomoteru Kamimura, Yutaka Aoyagi

**Affiliations:** Division of Gastroenterology and Hepatology, Graduate School of Medical and Dental Sciences, Niigata University, 1-757 Asahimachido-ri, Chuo-ku, Niigata, 951-8510 Japan; Department of Obstetrics and Gynecology, Niigata University Medical and Dental Hospital, 1-754 Asahimachido-ri, Chuo-ku, Niigata, 951-8510 Japan; General Center for Perinatal, Maternal and Neonatal Medicine, Niigata University Hospital of Medical and Dental Sciences, 1-754 Asahimachido-ri, Chuo-ku, Niigata, 951-8520 Japan; Department of Molecular Genetics, Graduate School of Medical and Dental Sciences, Niigata University, 1-757 Asahimachido-ri, Chuo-ku, Niigata, 951-8510 Japan; Department of Gastroenterology and Hepatology, Saiseikai Niigata Daini Hospital, 280-7 Teraji, Nishi-ku, Niigata, 950-1104 Japan

**Keywords:** Intrahepatic cholestasis of pregnancy, Bile acid, Ursodeoxycholic acid, Single-nucleotide polymorphism, *ABCB11*, *ABCB4*

## Abstract

**Background:**

Intrahepatic cholestasis of pregnancy (ICP) is a cholestasis condition caused by elevated levels of serum bile acids that mainly occurs in the third trimester of pregnancy. Maternal symptoms include pruritus; elevation of transaminases, biliary enzymes, and bilirubin levels; and abnormal liver function tests. Fetal symptoms include spontaneous preterm labor, fetal distress, and intrauterine death. It is more prevalent in the Caucasians and is rarely found in Asian countries, including Japan. The etiology of ICP has been reported as involving various factors such as, environmental factors, hormone balance, and genetic components. The genetic factors include single-nucleotide polymorphisms (SNPs) in the genes of canalicular transporters, including *ABCB4* and *ABCB11*. It has also been reported that the combination of these SNPs induces severe cholestasis and liver dysfunction.

**Case presentation:**

Here, we report for the first time a 24-year Japanese case of severe ICP diagnosed by typical symptoms, serum biochemical analysis, and treated with the administration of ursodeoxycholic acid which improved cholestasis and liver injury and prevented fetal death. The sequence analysis showed SNPs reported their association with ICP in the *ABCB11* (rs2287622, V444A) and *ABCB4* (rs1202283, N168N) loci.

**Conclusion:**

The risk of ICP has been reported to be population-specific, and it is rare in the Japanese population. Our case was successfully treated with ursodeoxycholic acid and the genetic sequence analysis has supported the diagnosis. Because genetic variation in *ABCB4* and *ABCB11* has also been reported in the Japanese population, we need to be aware of potential ICP cases in pregnant Japanese women although further studies are necessary.

## Background

The etiology of intrahepatic cholestasis of pregnancy (ICP) involves various factors such as environmental factors, hormonal balance, and genetic components. The relation of the genetic factors lead to the various prevalence rates among different ethnic groups. It has been reported that the prevalence in Caucasians in Europe, United States, Canada, and Australia ranges from 0.1% to 1.5% [1]. On the other hand, much fewer number of cases have been reported in Asian countries [2]. Symptoms include itching, particularly of the palms and feet, and jaundice. Fetal consequences of ICP include spontaneous preterm labor, fetal distress, and intrauterine death due to increased levels of serum bile acids [1–5]. Biochemical analysis shows a mild increase in transaminases, bilirubin, other biliary enzymes, and serum bile acids levels of >10 μmol/L [6, 7]. In addition, the level of cytotoxic bile acids, such as chenodeoxycholic acid, increases 10-100 times higher than the normal level. Symptoms and serum abnormalities abate after delivery; however, recently, reviews have reported that ICP is also related to an increased risk of developing hepatobiliary diseases later in life [8]. More recently, results of comprehensive analysis of genetic variation in ICP have been reported, and common variations around *ABCB4* and *ABCB11* encoding multidrug resistance protein 3 (MDR3) and bile salt export pump (BSEP), respectively, have been reported as key factors [1, 6]. Similar results have been reported that *ABCB4* [9–13] and *ABCB11* [14] have significant relationships with the etiology of ICP. The combination of these mutations has been reported to be related to severe ICP. For example, the combination of homozygous polymorphisms in *ABCB11* at the complementary DNA position 1331 with a thymine replaced by a cytosine (1331 T > C, rs2287622), leading to an exchange from valine to alanine (V444A), and in *ABCB4* at position 959 in exon 9 with a cytosine replaced by a thymine (959 C > T), leading to an exchange of serine to phenylalanine (S320F), and some other synonymous SNPs are considered to be related to the etiology of severe type of ICP [15]. Further analysis has shown that the association of 1331 T > C (rs2287622) was also driven by other SNP (rs3815676) [1]. Therefore, these genetic variations may contribute to the difference of the occurrence in ethnic groups and further studies will clarify the more accurate contributions of each variation, since these SNPs have also been found in general population [16–18]. Here, we report the first case of a Japanese woman diagnosed with ICP based on severe pruritis, increased levels of bile acid and hepatobiliary enzymes, and successfully treated with ursodeoxycholic acid (UDCA) preventing fetal death and clinical symptoms as well. The genetic sequence analysis showed a homozygous polymorphism in *ABCB11* at 1331 T > C leading to V444A that is often reported in the conditions [1, 15]. Furthermore, another synonymous single-nucleotide polymorphism in *ABCB4* (504 C > T, rs1202283, N168N) was combined in our patient that have never been reported the association with this disease. Based on these results, we concluded our case as a first Japanese case of severe ICP with mutations in *ABCB11* and *ABCB4*. Further study will help us to find and appropriately treat patients preventing fetal death and clinical symptoms.

## Case presentation

**Figure 1:**
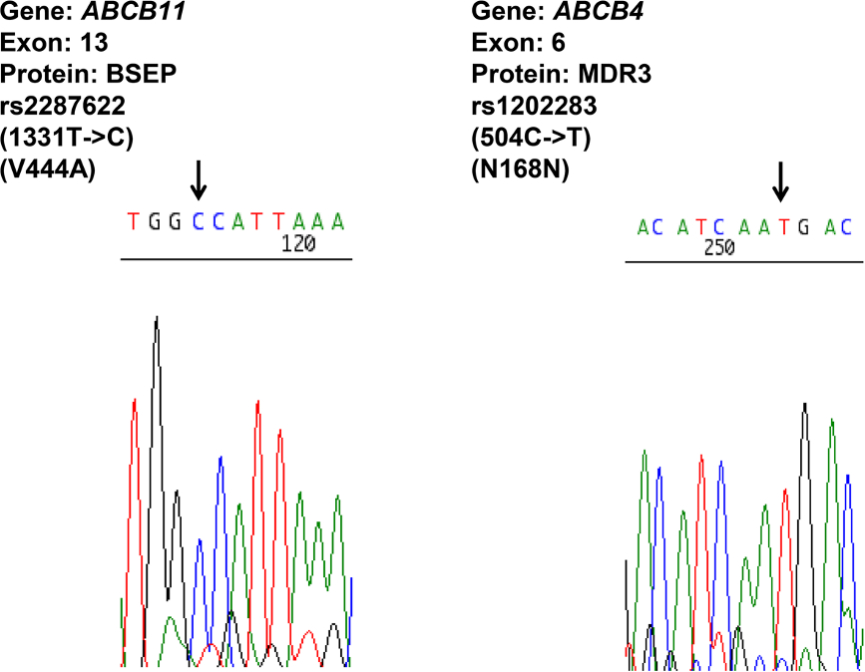
**Single-nucleotide polymorphisms in** ***ABCB11*** **and** ***ABCB4*** **of the patient.** Sequencing of all the coding exons of *ABCB4* and *ABCB11* was performed with the standard genomic sequencing procedure. Black arrows indicate the mutations found in the patient. Genetic information from the database is shown in the upper panel.

A 24-year-old Japanese pregnant female presented with severe pruritis and jaundice in the 15th week of her first pregnancy and was referred to our department. She had never been diagnosed with a constitutional hyperbilirubinemia. Physical examination revealed skin and conjunctival jaundice. No nausea, vomiting, headache, or fatigue were noted, and her blood pressure and other physiological tests were normal. Laboratory tests revealed the increased levels of direct bilirubin (D-Bil, 4.0 mg/dl), aspartate aminotransferase (AST, 264 IU/l), alanine aminotransferase (ALT, 545 IU/l), and lactate dehydrogenase (LDH, 241 IU/l). Total bile acid level was high (89.6 μmol/l), and the ratio of the cytotoxic chenodeoxycholic acid to total bile acid (C/BA) was 0.24 at the time of admission (Table 1). No hemolysis or decrease in platelet count was observed, and tests for viral markers including hepatitis A, B, and C and autoimmune markers including anti-nuclear, anti-mitochondrial, and anti-phospholipid antibodies were negative. Slight increases in AFP (α-fetoprotein) to 36 ng/ml and AFP-L3 (Lens culinaris agglutinin -reactive α-fetoprotein isoform) to 32.8%, which are often found in pregnancy were detected and protein induced by Vitamin K absence or antagonists-II (PIVKAII) showed normal level (Table 1). No proteinuria or ketonuria was noted on urine tests. Ultrasonography revealed no chronic damage or acute fatty infiltration in the liver. Computed tomography was not utilized due to the pregnancy. The patient’s mother had a history of third-trimester pruritis; however, her symptoms were milder, and she had no history of fetal death or preterm delivery. No symptoms were marked in her grandmother. Based on these findings, she was diagnosed with ICP with severe clinical symptoms, although rare in the Japanese population. To support the diagnosis, we obtained the patient’s written informed consent and performed genomic sequencing of genes such as *ABCB4* and *ABCB11*, the genetic variations of which have been reported to be related to the etiology and severity of the disease (approved by the ethics committee at the Niigata University, No. 536). Sequencing of all the coding exons of the genes revealed a nonsynonymous homozygous mutation in *ABCB11* and a synonymous mutation in *ABCB4* (Figure 1). The nonsynonymous polymorphism in *ABCB11* was a major single-nucleotide exchange from thymine to cytosine at the position 1331 (1331 T > C), leading to an exchange from valine to alanine (V444A). This has been reported to be strongly associated with ICP based on a large cohort study [1]. The synonymous mutation in *ABCB4* was 504 C > T, which has never been reported to be association with the disease.

**Table 1:** **Results of laboratory investigation**

**Hematology**			**Biochemistry**		
WBC	10,080 /μl	TP	6.8 g/dl	TG	197 mg/dl
RBC	410x10^4^ /μl	Alb	3.7 g/dl	TC	191 mg/dl
Hb	13.3 g/dl	BUN	8 mg/dl	IgG	908 mg/dl
Ht	39.8%	Cre	0.36 mg/dl	CRP	0.05 mg/dl
PLT	25.5x10^4^ /μl	T-Bil	6.3 mg/dl	Bile acid	89.6 μmol/l
		D-Bil	4.0 mg/dl	C/BA	0.24
**Coagulation**		AST	264 IU/l	HbA1c	4.2%
PT%	121%	ALT	545 IU/l	**Serum Marker**	
		ALP	310 IU/l	AFP	36 ng/ml
		LDH	241 IU/l	AFP-L3	32.8%
		γ-GTP	14 IU/l	PIVKAII	44 mAU/ml
		ChE	137 IU/l	ANA	(-)
				AMA	(-)

### Clinical course

**Figure 2:**
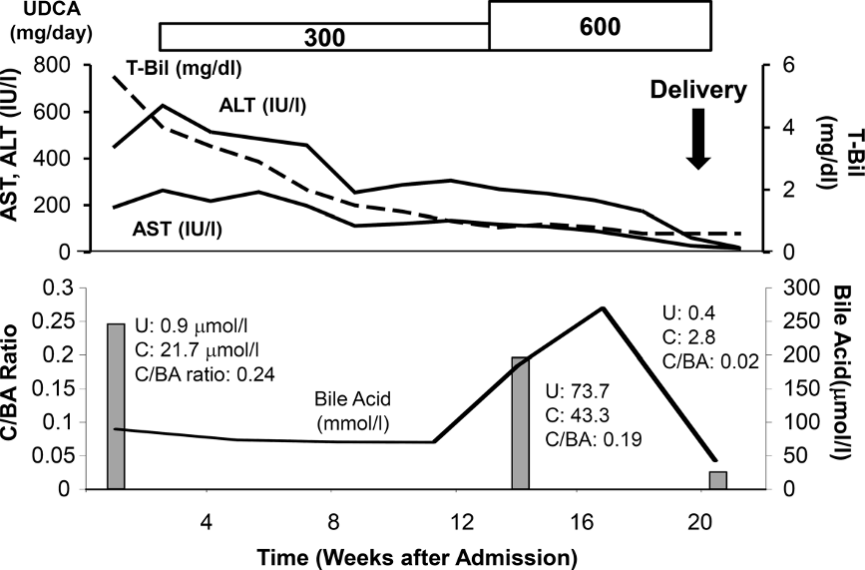
**Clinical course of the patient.** The time-dependent levels of AST, ALT, and T-Bil are shown in the upper panel. The gray bar indicates the ratio of chenodeoxycholic acid in bile acid (C/BA ratio), and the black line in the lower panel indicates the total bile acid level. Ursodeoxycholic acid, U; Chenodeoxycholic acid, C; Bile acid, C/BA.

After the diagnosis of ICP, the patient was treated with UDCA (300 mg daily) from the 18th day of her admission and the dose was increased to 600 mg daily at 13 weeks after the admission since the level of ALT showed mild increasing pattern (Figure 2). Her pruritis slowly improved, and D-Bil, AST, ALT, and LDH levels slowly decreased to normal ranges after the initiation of the administration of UDCA and returned to the normal levels of 0.6 mg/dl, 17 IU/l, 20 IU/l, and 135 IU/l, respectively within 3 weeks after the spontaneous preterm delivery of a healthy and well-developed infant at the 35th week of gestation (Figure 2). Her C/BA ratio decreased to 0.19 (total bile acid level of 270.3 μmol/l) within 12 weeks and returned to the minimum level of 0.02 (total bile acid level of 42.5 μmol/l) just after delivery. The administration of UDCA was discontinued 3 weeks after delivery, and no symptom recurrence or increases in the level of hepatobiliary enzymes were observed.

## Discussion

Viral hepatitis, autoimmune liver injury, hyperemesis gravidarum, acute fatty liver of pregnancy, HELLP (hemolysis, elevated liver enzyme levels, and low platelet levels) syndrome, preeclampsia, and ICP are known to be involved in the etiology of liver injury found during pregnancy [1, 6]. Among these diseases, ICP is frequently found in Caucasians, and the difference in the prevalence rate of ICP among the populations is due to genetic variation in the genes encoding ATP-Binding Cassette (ABC) transporters [2]. The most common symptom is pruritis, which typically presents in the third trimester of pregnancy, but early disease onset can be observed in ~20% of cases [7]. Symptoms disappear after delivery; however, it has been reported that ICP is associated with an increased risk of developing various hepatobiliary diseases, including gallstones and cirrhosis, later in life [8]. Biochemical analysis revealed increased levels of transaminases, bilirubin, and serum bile acids (>10 μmol/l). However, clinical jaundice is detected in 10%-15% of patients, and bile acid levels rarely exceed 100 μmol/l [2, 5]. The symptoms and the level of serum enzymes ranged variously, and the severe type was reported with an early onset, severe pruritis, significant elevation of the hepatobiliary enzymes [15].

Recent large cohort studies and reviews reported that common variations around *ABCB4* and *ABCB11* as key factors for developing ICP [1–6]. In addition, it has been reported that the SNP in *ABCB11* (1331 T > C), leading to an exchange from valine to alanine (V444A), is related to severe type of ICP [14]. These genetic backgrounds might affect the expression of transporters and their structures that transport sulphated progesterone metabolites which significantly increases during pregnancy leading to the clinical symptoms [19]. Our case showed significant pruritis and increased levels of total and cytotoxic bile acids at the time of admission that was first trimester of pregnancy. Her serum markers showed no evidence of other hepatic diseases, such as HELLP syndrome, acute fatty liver of pregnancy, autoimmune liver diseases, or viral chronic hepatitis. The major SNP in *ABCB11* (1331 T > C, rs2287622) and the additional synonymous mutation in *ABCB4* (504 C > T, rs1202283) were confirmed by the genomic sequencing strategy. These results are indicating the potential relationship with the disease [14] and its severity [15], however since these variations have also been found in general population [16–18], the diagnosis of the disease based on the clinical symptoms and exclusion of other liver diseases like our case. Although a standard therapeutic method has not been established, two recent studies encouraged the administration of UDCA for ICP to reduce pruritis and improve hepatobiliary enzyme levels to reduce the risk of fetal death [20, 21]. The therapeutic effect of UDCA has been reported from various aspects. It replaces cytotoxic bile acid, such as chenodeoxycholic acid into UDCA, directly. In addition, it increases the membrane transport expression, such as MDR3 and BSEP, leading to the stimulation of hepatobiliary excretion of progesterone disulphates that ameliorate pruritus and liver injury [19]. These effects contribute for the treatment of ICP [20, 21]. In our case, UDCA was effective for improving symptoms, i.e., led to decreases in levels of hepatobiliary enzymes and the C/BA ratio, and prevented intrauterine death and meconium passage. The C/BA ratio was used to define the level of bile acid toxicity because the total bile acid level increased after the administration of UDCA followed by replacement with cytotoxic chenodeoxycholic acid. Further studies will help the understandings and the genetic diagnosis, because no need exists to perform liver biopsy as an interventional method to diagnose the disease. In addition, as it is also known that ICP recurs in subsequent pregnancy [2], it is important to be aware of the disease and its therapeutic options.

## Conclusion

The risk of ICP has been reported to be population-specific, and it is rare in the Japanese population. Here, we report the first Japanese case of ICP with severe symptoms and successfully treated with UDCA. The genetic sequence analysis also supported the diagnosis. Because genetic variation in *ABCB4* and *ABCB11* has also been reported in the Japanese population, we need to be aware of potential ICP cases in pregnant Japanese women although further studies are necessary.

### Consent

Written informed consent was obtained from the patient for publication of this case report and any accompanying images.
